# Polymorphisms in the Osteopontin Are Associated with Susceptibility to Ankylosing Spondylitis in a Han Chinese Population

**DOI:** 10.1155/2018/3458439

**Published:** 2018-01-18

**Authors:** Juyi Li, Yi Cai, Zhongjing Wang, Aiping Deng, Guoliang Yang

**Affiliations:** ^1^Department of Pharmacy, The Central Hospital of Wuhan, Tongji Medical College, Huazhong University of Science and Technology, Wuhan, Hubei, China; ^2^Department of Pain, The Central Hospital of Wuhan, Tongji Medical College, Huazhong University of Science and Technology, Wuhan, Hubei, China; ^3^Department of Endocrinology, The Central Hospital of Wuhan, Tongji Medical College, Huazhong University of Science and Technology, Wuhan, Hubei, China; ^4^Department of Information, The Central Hospital of Wuhan, Tongji Medical College, Huazhong University of Science and Technology, Wuhan, Hubei, China

## Abstract

The aim of this study was to investigate whether osteopontin* (OPN)* variants are associated with susceptibility to ankylosing spondylitis (AS) in a Chinese population. Polymorphisms at the 9175th position in exon 7 of* OPN* and rs17524488 were genotyped using direct sequencing in 186 unrelated AS patients and 188 ethnically matched healthy controls. Serum concentration of OPN was measured by enzyme-linked immunosorbent assay (ELISA) in all participants. AS patients displayed significantly higher OPN serum levels than the controls (*P* < 001). A heterozygous, novel 9175 T>A in exon 7 of the* OPN* gene was found in this study. In healthy controls, subjects carrying the rs17524488 G/G genotype of the* OPN* display significantly higher OPN serum levels than the GG/GG genotype (*P* < 0.05). Plasma OPN level is implicated as an early diagnostic marker of AS. The novel 9175th- (exon 7) position polymorphism of* OPN *and rs17524488 were related to susceptibility to AS in a Chinese population, the rs17524488 G/G genotype may be involved in the pathogenesis of AS, and the precise molecular mechanism underlying the influence of* OPN* polymorphisms on the development of AS remains to be determined in the further prospective studies.

## 1. Introduction

Ankylosing spondylitis (AS) is an autoimmune disease caused by chronic inflammation response and pathological mineralization and usually strikes the males [1, 2]. Incipient symptoms of AS appear before 40 years of and it is characterized by inflammatory back pain and the stiffness and ankylosis of spinal joints [3]. Approximately 0.2–1.4% of the general population suffers from AS, and its incidence is 0.2–0.54% in the Chinese [2, 4].

AS is highly heritable and many genetic polymorphisms have been reported to correlate the onset of AS [5].* HLA B27* has been reported to be as a major genetic contributor to AS and 90% AS patients are accompanied with the positive HLA B27 [6]; in addition, there are other genetic polymorphisms, such as* JMY*,* PTGER4*,* JARID1A*, and* ANTXR2* [7, 8]. Although many studies focus on the pathogenesis of AS, the etiology and mechanisms behind AS remain unclear.

Osteopontin (OPN) is a secreted phosphoglycoprotein with several functions in different physiological and pathological processes, including bone remodeling process, inflammation, autoimmune responses, and tumorigenesis [9]. Studies have shown that OPN serum levels are higher in some kinds of autoimmune diseases than healthy controls and may influence development of these diseases through enhancing the proinflammatory T helper type 1 (TH1) and TH17 cell responses and inhibiting the TH2 responses [10]. More than 10 SNPs have been identified in the* OPN* promoter. These polymorphisms may affect the transcriptional activity of* OPN* and some of them are thought to be genetic risk factors for disease susceptibility [11–13], of which the rs17524488 (-156 GG/G) polymorphism is most frequently studied, which has been found to associate with several diseases, including hip osteoarthritis [14], cancer [15], and diabetic nephropathy [16].

These observations led us to hypothesize that OPN polymorphisms may be involved in the pathogenesis of AS through inflammation and/or the bone remodeling process. However, there have been little studies investigating the association of the* OPN *polymorphisms and AS to date. Therefore, the aim of this study was to investigate if an association exists between* OPN* polymorphisms, OPN serum levels, and the risk of AS in a Chinese population.

## 2. Materials and Methods

### 2.1. Subjects

From May 2010 to October 2013, 188 healthy volunteers for physical examination and 186 unrelated AS patients were recruited from the Affiliated Hospital of Ningxia Medical University in a Han Chinese population. All AS patients are HLA-B27 positive. Patients diagnosed with AS according to the modified New York criteria developed in 1984 were included in the study. All of the subjects gave written informed consent. This study was approved by the Ethics Committee of Ningxia Medical University. Two mL fasting venous blood was collected from all subjects; one mL full blood sample was used to extract genomic DNA; however, the other one mL full blood sample was centrifuged (10 minutes at 1160*g*) to separate the clot material from the solution phase (serum) which was stored at −80°C for further analysis. Genomic DNA was extracted using TIANGEN reagent set (Beijing) following standard protocols; DNA samples were stored at −20°C. Exclusion criteria were patients with diabetes mellitus, cancer, severe liver and kidney failure, and being on therapy for any chronic inflammatory disease. Healthy volunteers as the control group were recruited from the department of physical examination at the same time, and those carrying HLA-B (rs13202464) were excluded.

### 2.2. Information Collection

The following information was collected: sex, age, duration of disease, body mass index (BMI), smoking status, family history of AS, and HLA-B27. The Bath Ankylosing Spondylitis Disease Activity Index (BASDAI), Bath Ankylosing Spondylitis Functional Index (BASFI), and Bath Ankylosing Spondylitis Global (BAS-G) score were applied to evaluate the disease activity, physical function, and global wellbeing, respectively. The modified Chinese versions of BASDAI, BASFI, and BAS-G have good intraclass correlation and Cronbach's alpha [17].

### 2.3. Analysis of Polymorphisms in the OPN Regulatory Region

The 9175th position in exon 7 of* OPN* and rs17524488 variants were genotyped by direct sequencing of the sense and antisense strands following polymerase chain reaction (PCR) amplification. A primer pair of the 9175th position was 5′-TACCCTGATGCTACAGACGAGG-3 (forward) and 5′-CTGACTATCAATCACATCGGAATG-3′ (reverse). A primer pair of rs17524488 was 5′-TGTCACTAGTGCCATTTGT-3′ (forward) and 5′-TGTACCTTGGTCGGCGTTTG-3′ (reverse). PCR was performed using 50 ng DNA as a template under the following conditions: 95°C for 3 min, then 35 cycles of 94°C for 30 s, an annealing temperature at 60°C for 45 s, and 72°C for 30 s, with a final extension at 72°C for 10 min. The PCR products were direct sequencing using an automated ABI 3100 DNA sequencer by GeneCore BioTechnologies (Shanghai, China).

### 2.4. OPN ELISA Assay

Serum concentration of OPN was measured by ELISA according to the protocol provided by the manufacturer (Raybiotech, Norcross, USA) in all participants. Serum was diluted as 1 : 10 into sample diluents. The optical density was measured at 450 nm (BioRad, USA).

### 2.5. Statistical Analysis

Statistical analysis was performed using SPSS 15.0 software. One way ANOVA and *t*-test were used to compare mean differences for continuous variables. Allele frequency was determined via direct counting. Binary logistic regression analysis was used to assess independent predictors of AS. Receiver operating characteristic curve (ROC) analysis was used to find the cut-off point of OPN for predicting AS. Differences in the distribution of genotypes between AS patients and control subjects were examined using the *χ*^2^ test, and a statistically significant difference was defined as *P* < 0.05.

## 3. Results

### 3.1. Subject Characteristics

As was shown in Table 1, a total of 374 subjects were enrolled in this study, containing 186 cases and 188 controls. The clinical characteristics such as age, sex, body mass index (BMI), and smoking status had no significant difference between the case and control groups (all *P* > 0.05). Family history has significant difference between the case and control groups (*P* < 0.05). In AS subjects, all patients were HLA-B27 positive and their mean disease duration, mean BASDAI, mean BASFI, and mean BAS-G scores were 5.67 ± 0.28, 4.23 ± 0.06, 2.79 ± 0.06, and 4.38 ± 0.09, respectively.

### 3.2. Association of* OPN* Genetic Polymorphisms with the Disease Activity Index in AS Patients

We analyzed the relationship between disease activity index (BASDAI, BASFI, and BAS-G) and the two polymorphisms of* OPN* in AS patients. No significant association between* OPN* polymorphisms and BASDAI, BASFI, or BAS-G (all *P* > 0.05) was found. However, we failed to improve the significance even after adjustment for the effects of age, sex, BMI, smoking status, and family history (Table 2).

### 3.3. Increased Levels of OPN in AS Patients and Diagnostic Value of OPN for AS

**Figure 1:**
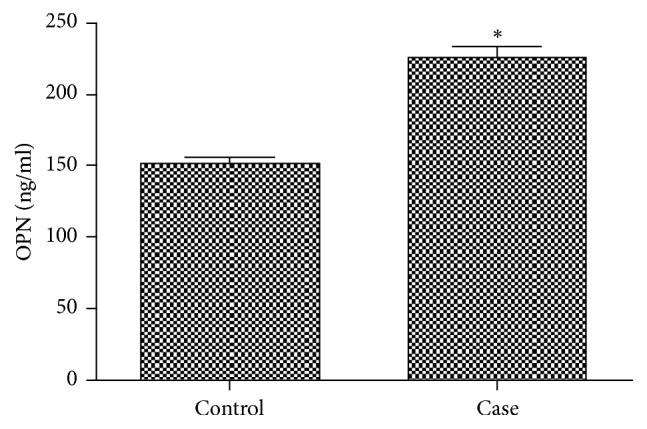
*Serum concentration of OPN in AS patients and healthy controls*. Compared with healthy controls, the serum levels of OPN in AS patients were significantly elevated (^*∗*^*P* < 0.01).

Serum concentration of OPN was measured in 186 AS patients and 188 ethnically matched healthy controls by ELISA, even after adjusting for confounding risk factors (age, sex, BMI, smoking status, and family history), which shows that AS patients displayed significantly higher OPN serum levels (median, 217.76; range, 102.02–468.65 ng/mL) than the controls (median, 156.99; range, 51.56–321.54 ng/mL) in Figure 1 and Table 3 (*P* < 001).

**Figure 2:**
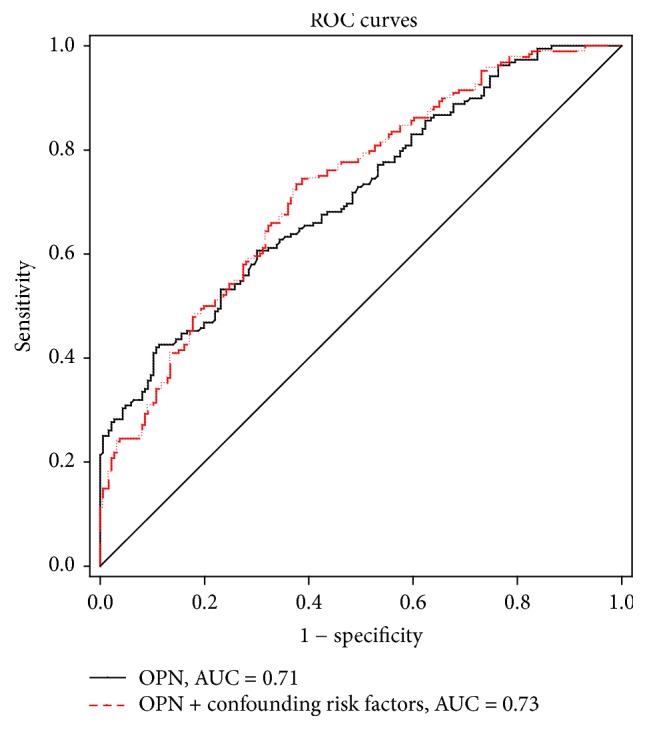
*Analysis of ROC curve to detect OPN in AS patients*. In ROC analysis, the AUC of Siglec-5 was 0.71; the AUC of confounding risk factors was 0.73.

Taking AS as the dependent variable, the risk factors (gender, age, BMI, family history, and smoking status) were entered into binary logistic regression analysis. After adjusting for the risk factors, plasma levels of OPN remained with a significant association with an increased odds ratio (OR) for AS (*P* < 0.001). ROC curve analysis was performed to verify the diagnostic accuracy of OPN for AS. The area under curve (AUC) of OPN was 0.71 (95% confidence intervals (CI) 0.662–0.764, *P* < 0.001) and the optimal cut-off point for siglec-5 was 131.7 ng/mL. At this level, the Youden index = 0.313, sensitivity was 42.2% (95% CI 0.349–0.494); specificity was 89.25% (95% CI 0.839–0.933). The AUC of OPN + confounding risk factors was 0.73 (95% CI 0.676–0.776, *P* < 0.001), which was higher than that of OPN, but this did not reach the level of statistical significance (*P* > 0.05) (Figure 2).

### 3.4. OPN Polymorphisms Are Associated with AS Patients

**Figure 3:**
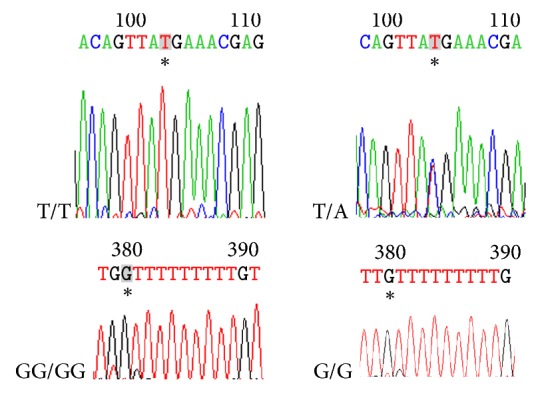
The results of DNA sequencing.

DNA fragments from 9041 to 9292 in exon 7 of the* OPN* gene and one known polymorphism (rs17524488) were analyzed using direct sequencing in 186 AS patients and 188 ethnically matched healthy controls. We found a heterozygous, novel 9175 T>A in exon 7 of the* OPN* gene, and there is a small insertion at nt-156 (rs17524488), which has only two alleles: G/G and GG/GG. Overall distributions of genotypes of* OPN* gene 9175 and rs17524488 were significantly different in AS patients controls (all *P* < 0.005). Frequencies of allele A and allele G were all higher in AS patients than in the controls (all *P* < 0.005), indicating that subjects who carried allele A or allele G have a significantly higher risk of developing AS (Table 4). The results of DNA sequencing were shown in Figure 3.

### 3.5. OPN Polymorphisms Are Associated with the OPN Levels

**Figure 4:**
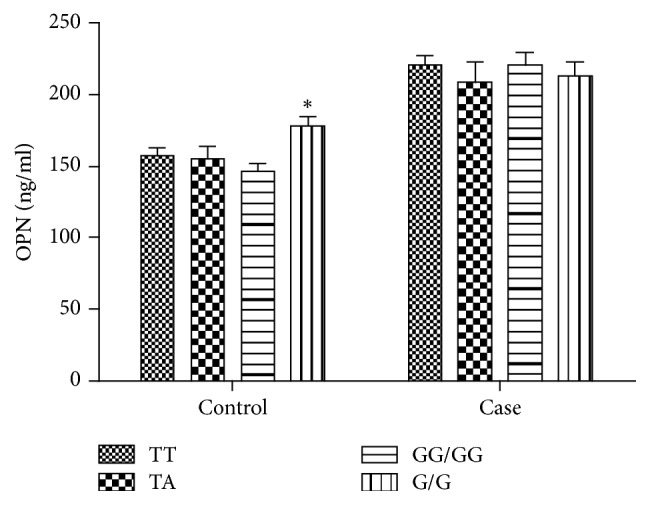
*OPN polymorphisms are associated with the OPN levels*. In healthy controls, subjects carrying the rs17524488 G/G genotype of the* OPN* display significantly higher OPN serum levels than the GG/GG genotype (^*∗*^*P* < 0.05).

As shown in Figure 4, subjects carrying the rs17524488 G/G genotype of the* OPN* display significantly higher OPN serum levels than the GG/GG genotype (^*∗*^*P* < 0.05), but there were no significant associations between TT or TA genotypes with OPN serum levels in healthy controls. However, there were no significant associations between* OPN* genotypes and OPN serum levels in AS patients.

## 4. Discussion

In this study, a heterozygous, novel 9175 T>A in exon 7 of the* OPN* gene and rs17524488 were significantly associated with a genetic predisposition for AS. Patients with AS had higher serum levels of OPN compared with controls, even after adjusting for confounding risk factors. However, the rs17524488 G/G genotype of the* OPN* was significantly correlated with an increased OPN serum level compared to the GG/GG genotype in healthy controls, which suggests that rs17524488 G/G genotype may be involved in the pathogenesis of AS. This is the first study to demonstrate a strong relationship between the 9175th- (exon 7) position polymorphism of* OPN *or rs17524488 and AS patients.

We first considered the possibility that* OPN* polymorphisms are related to the inflammatory process in AS. It has been reported that patients with AS had significantly higher plasma OPN, TNF-alpha, and IL-6 levels and more mRNA expression than healthy controls. The plasma OPN level was correlated with serum ALP, OCN, and CTX-I levels, but not with disease activity in AS. OPN might be involved in bone remodeling rather than in inflammation in AS [18]. In this study, no significant association between* OPN* polymorphisms and disease activity index (all *P* > 0.05) was found, and we also found that patients with AS had significantly higher plasma OPN than healthy controls which is consistent with them [18]. We found a heterozygous, novel 9175 T>A in exon 7 of the* OPN* gene, and distributions of genotypes of* OPN* gene 9175 and rs17524488 were significantly different in AS patients controls. Frequencies of allele A and allele G were all higher in AS patients than in the controls, indicating that subjects who carried allele A or allele G have a significantly higher risk of developing AS (OR = 1.853 or OR = 1.327). In addition, subjects carrying the rs17524488 G/G genotype of the* OPN* display significantly higher OPN serum levels than the GG/GG genotype in healthy controls, strongly indicating that the rs17524488 G/G genotype of the* OPN *may be involved in the pathogenesis of AS. Thus, the mechanism by which this polymorphism contributes to AS susceptibility is less clear. There is evidence suggesting that OPN acts as a proinflammatory cytokine and plays an important role in regulating inflammation [18]; subjects carrying polymorphism loci of the* OPN* will be more sensitive to the immune response; in addition, it might be relevant in the regulation of OPN production in response to the initial immunostimulating trigger.

So far, genetic variants in the* OPN* gene have shown being involved in susceptibility to other immune-mediated diseases such as systemic lupus erythematosus [19], Crohn's disease [20], rheumatoid arthritis [21], and lupus nephritis [22]. In addition, overexpression of OPN has been described in several basic inflammatory processes, such as arthritis [23], myocardial remodeling after infarction [24], kidney interstitial fibrosis after obstructive uropathy [25], wound healing [26], and several types of cancer [27], where it is demonstrated that* OPN* gene polymorphism and OPN levels play important roles in immune- and inflammatory-mediated diseases, including AS.

There are some limitations of the present study that should be considered. Large sample study needs to be explored further. Although this case-control study revealed a strong relationship between the 9175th- (exon 7) position polymorphism of* OPN *or rs17524488 and AS patients, the precise molecular mechanism underlying the influence of* OPN* polymorphisms on the development of AS remains to be determined in further prospective studies.

## 5. Conclusions

In conclusion, plasma OPN level is implicated as an early diagnostic marker of AS; the novel 9175th- (exon 7) position polymorphism of* OPN *or rs17524488 was related to susceptibility to AS in a Chinese population; the rs17524488 G/G genotype may be involved in the pathogenesis of AS, and the precise molecular mechanism underlying the influence of* OPN* polymorphisms on the development of AS remains to be determined in further prospective studies.

## Figures and Tables

**Table 1:** Characteristics of AS patients and healthy control (mean ± SEM).

Variable	Case (%)	Control (%)	*P*
Gender			
Male	96 (51.61)	100 (53.19)	0.760
Female	90 (48.39)	88 (46.81)	
Age (years)	27.24 ± 0.68	28.90 ± 0.68	0.084
BMI (kg/m^2^)	24.68 ± 0.29	25.17 ± 0.21	0.166
Disease duration (years)	5.67 ± 0.28	—	—
BASDAI (0–10)	4.23 ± 0.06	—	—
BASFI (0–10)	2.79 ± 0.06	—	—
BAS-G (0–10)	4.38 ± 0.09	—	—
Family history			
Yes	25 (13.44)	12 (6.38)	*0.022*
No	161 (86.56)	176 (93.62)	
Smoking status			
Yes	85 (45.70)	92 (48.94)	0.531
No	101 (54.30)	96 (51.06)	

Significant values are written in italics.

**Table 2:** Difference in the scores of BASDAI, BASFI, and BAS-G among AS patients stratified by different AS genotype.

SNP	Genotype	Number (%)	BASDAI	BASFI	BAS-G
9175 (exon 7)	TT	142 (76.34)	4.21 ± 0.07	2.79 ± 0.07	4.34 ± 0.10
	TA	44 (23.66)	4.30 ± 0.12	2.80 ± 0.12	4.50 ± 0.18
	Unadjusted *P* value		0.561	0.965	0.422
	Adjusted *P* value		0.614	0.795	0.334

rs17524488	GG/GG	102 (54.84)	4.20 ± 0.08	2.79 ± 0.08	4.37 ± 0.12
	G/G	84 (45.16)	4.27 ± 0.09	2.79 ± 0.11	4.38 ± 0.13
	Unadjusted *P* value		0.529	0.948	0.961
	Adjusted *P* value		0.545	0.988	0.821

Data represent means ± SEM, adjusted for the effects of age, sex, BMI, family history, smoking status, and disease duration.

**Table 3:** Risk factors for AS by binary logistic regression analysis.

	OR	95% CL for OR	*P*	OR^*∗*^	95% CL for OR^*∗*^	*P* ^*∗*^
Sex	1.065	0.710–1.599	0.760	/	/	/
Age	0.981	0.959–1.003	0.085	/	/	/
BMI	0.959	0.904–1.018	0.167	/	/	/
Family history	2.277	1.108–4.682	*0.025*	/	/	/
Smoking status	0.878	0.585–1.318	0.531	/	/	/
OPN	1.011	1.007–1.014	*0.000*	1.011	1.007–1.014	*0.000*

CI, confidence interval. Logistic regression models were used to calculate OR. ^*∗*^Adjusted for gender, age, BMI, family history, and smoking status. Significant values are written in italics.

**Table 4:** Frequencies of genotypes and alleles of *OPN* in AS patients and healthy control.

Genotype/allele	Case (*n* = 186), *n* (%)	Control (*n* = 188), *n* (%)	OR (95% CL)	*P*
9175 (exon 7)				
Genotype				
TT	142 (76.34)	164 (87.23)	1.853 (1.177–2.918)	*0.006*
TA	44 (23.66)	24 (12.77)		
Allele				
T	328 (88.17)	352 (93.62)	1.853 (1.151–2.983)	*0.01*
A	44 (11.83)	24 (6.38)		

rs17524488				
Genotype				
GG/GG	102 (54.84)	124 (65.96)	1.327 (1.029–1.711)	*0.028*
G/G	84 (45.16)	64 (34.04)		
Allele				
GG	204 (54.84)	248 (65.96)	1.327 (1.108–1.588)	*0.002*
G	168 (45.16)	128 (34.04)		

Significant values are written in italics.
